# Oral granulomatosis with polyangiitis a systematic review

**DOI:** 10.1002/cre2.706

**Published:** 2023-01-04

**Authors:** Alberto J. Peraza Labrador, Luciano H. M. Valdez, Nestor R. Gonzalez Marin, Karem A. R. Ibazetta, Joan A. L. Chacón, Alberto J. V. Fernandez, Marcelo S. V. Valencia, Schillin W. Marchant, Katman B. T. Sanchez, Cesar A. Villacrez

**Affiliations:** ^1^ Centro de Odontologia Integral Acarigua Venezuela; ^2^ Department of Diagnostic Sciences Texas A&M University School of Dentistry Texas Estados Unidos; ^3^ Diagnocentrobucal Lima Peru; ^4^ Department of Otolaryngologist Military Hospital Bogota Colombia Bogotá Colombia; ^5^ Oral and Maxilofacial Pathology and Medicine Clinics Department Lima Peru; ^6^ Dental spa C.A. San Cristobal Tachira Venezuela; ^7^ Centro Médico Odontológico ODOMED Caracas Venezuela; ^8^ Oral Surgery Department, Dental School Universidad Internacional de Ecuador Quito Ecuador; ^9^ Doctoral Program in Morphological Sciences Universidad de la Frontera Temuco Chile; ^10^ Clinical Dental New Hope Lima Peru; ^11^ Dental Implant Department Universidad Señor de SIPAN Dental School Lima Peru

**Keywords:** antineutrophil cytoplasmic, gingivitis, granulomatosis with polyangiitis

## Abstract

**Objective:**

Granulomatosis with polyangiitis is an unusual multisystemic inflammatory disease, with vasculitis of small‐ and medium‐sized vessels, with a predilection for upper lower airways and kidneys. The etiology remains unknown although it may originate from different stimuli, in genetically susceptible patients.

**Materials and Methods:**

A detailed database search was performed. The variables were demographics, localization, histopathological findings, antineutrophil cytoplasmic autoantibody, cytoplasmic (c‐ANCA) tests, treatment, and follow‐up.

**Results:**

Fifty‐two cases were identified; the mean age was 49.6 years, with a range from 6 to 87 years. It was most frequently seen in females (57.7%). The most common race was white (59.6%). The most frequent location was in the maxillary gingiva (28.8%), followed by both the upper and lower gingiva (19.2%). The most common clinical presentation was “strawberry gingivitis” (61.5%). The main symptom was pain, in 50%. Regarding the c‐ANCA test, it was positive in 71.2% of cases. The most common therapy was prednisone and cyclophosphamide, utilized in 51.9%. The average follow‐up was 23.6 months, and 88.5% of patients were still alive at follow‐up.

**Conclusion:**

The diagnosis initially was difficult to establish, an early diagnosis and treatment are mandatory. If untreated the disease can be associated with morbidity and mortality. For the oral clinician, this disease needs to be addressed in the differential diagnosis of oral lesions.

## 1 INTRODUCTION

Granulomatosis with polyangiitis (GPA), formerly known as Wegener's granulomatosis (WG), is a systemic inflammatory disease (Patten & Tomecki, 1993). It was first described by Klinger (1931) and then by Wegener (1936). According to the 2012 International Chapel Hill Consensus Conference on the Nomenclature of Vasculitides, GPA is defined as a necrotizing granulomatous inflammation usually involving the upper and lower respiratory tract with necrotizing vasculitis affecting predominantly small to medium vessels (Jennette et al., 2013). The American College of Rheumatology established that the diagnosis of GPA can be made if two of the following criteria are present: ulcerative lesions in the oral mucosa or nasal bleeding and/or inflammation, nodules, fixed infiltrates, or cavities in a chest radiograph, abnormal urinary sediment, and granulomatous inflammation on biopsy (Stewart et al., 2007).

Regarding incidence, 23.7–156.5 cases per million have been reported, with an annual incidence of 3–14.4 cases per million (Almouhawis et al., 2013). In the United States, approximately 3 cases occur per 100,000 persons, and 5 per 100,000 persons in Europe (Greco et al., 2016). The head and neck are involved in nearly 90% of cases, with the nose, eyes, ears, and mouth the most often affected (Apoita‐Sanz et al., 2020). GPA usually begins as a localized process, which, if not diagnosed and treated, may progress at an unpredictable rate to involve several tissues or organs (Kertesz et al., 2021). Constitutional symptoms such as general malaise, myalgia, arthralgia, anorexia, weight loss, and pyrexia can be observed, but at the beginning, mild symptomatology is usually seen (Apoita‐Sanz et al., 2020). In this regard, the common manifestations of the disease include the classic triad of upper airways (87%), lung (69%), and kidney (48%) involvement (Hoffman et al., 1992).

Oral cavity manifestation is present in 6%–13% of the patients during the disease course, but oral involvement as the first sign of the disease is found in only 2% of the cases, representing an important diagnostic pitfall (Almouhawis et al., 2013; Ponniah et al., 2005). Initial oral cavity lesions may present as nonspecific erosive/ulcerative lesions or appear as hyperplastic gingivitis (Hoffman et al., 1992). Strawberry‐like gingivitis is the characteristic sign, manifesting as enlarged, erythematous interdental papillae containing red to purple petechiae and a granular appearance (Almouhawis et al., 2013; Apoita‐Sanz et al., 2020). The diagnosis is difficult, since the disease develops over an extended period of time, with 4.7–15 months from the beginning of the symptoms to the diagnosis (Bergé et al., 2000). A test that helps is antibodies to neutrophil cytoplasmic antigens (antineutrophil cytoplasmic autoantibody, cytoplasmic, c‐ANCA) which are present in about 80%–90% of patients and appear to play a role in pathogenesis, but are not likely to be essential to cause disease (Hoffman et al., 1992). There are 2 types of staining: cytoplasmic (c‐ANCA) and perinuclear (perinuclear antineutrophilic cytoplasmic antibodies, p‐ANCA). Most patients with a c‐ANCA pattern have an ANCA directed against proteinase‐3 (PR3), as determined by enzyme‐linked immunosorbent assay, while those with the p‐ANCA pattern usually have an ANCA directed against myeloperoxidase (Chen & Kallenberg, 2009).

The prognosis of uncontrolled GPA is poor, with most deaths resulting from renal failure secondary to glomerulonephritis (Eufinger et al., 1992). For that reason, corticoid therapy has been necessary to avoid deaths in the majority of cases (Ponniah et al., 2005). In this regard, a systematic review of reported cases, case series, and a prospective study of oral GPA was performed to determine presentation, diagnostic features, presented treatments, and patient outcomes.

## 2 MATERIALS AND METHODS

A systematic review of the published literature on cases of oral GPA was performed. According to the guidelines set forth by the Institutional Review Board of Centro de Odontologia Integral Acarigua Portuguesa state Venezuela, this study met the criteria for nonhuman subject research, and as a result board approval was not required.

### 2.1 Search strategy

**Figure 1:**
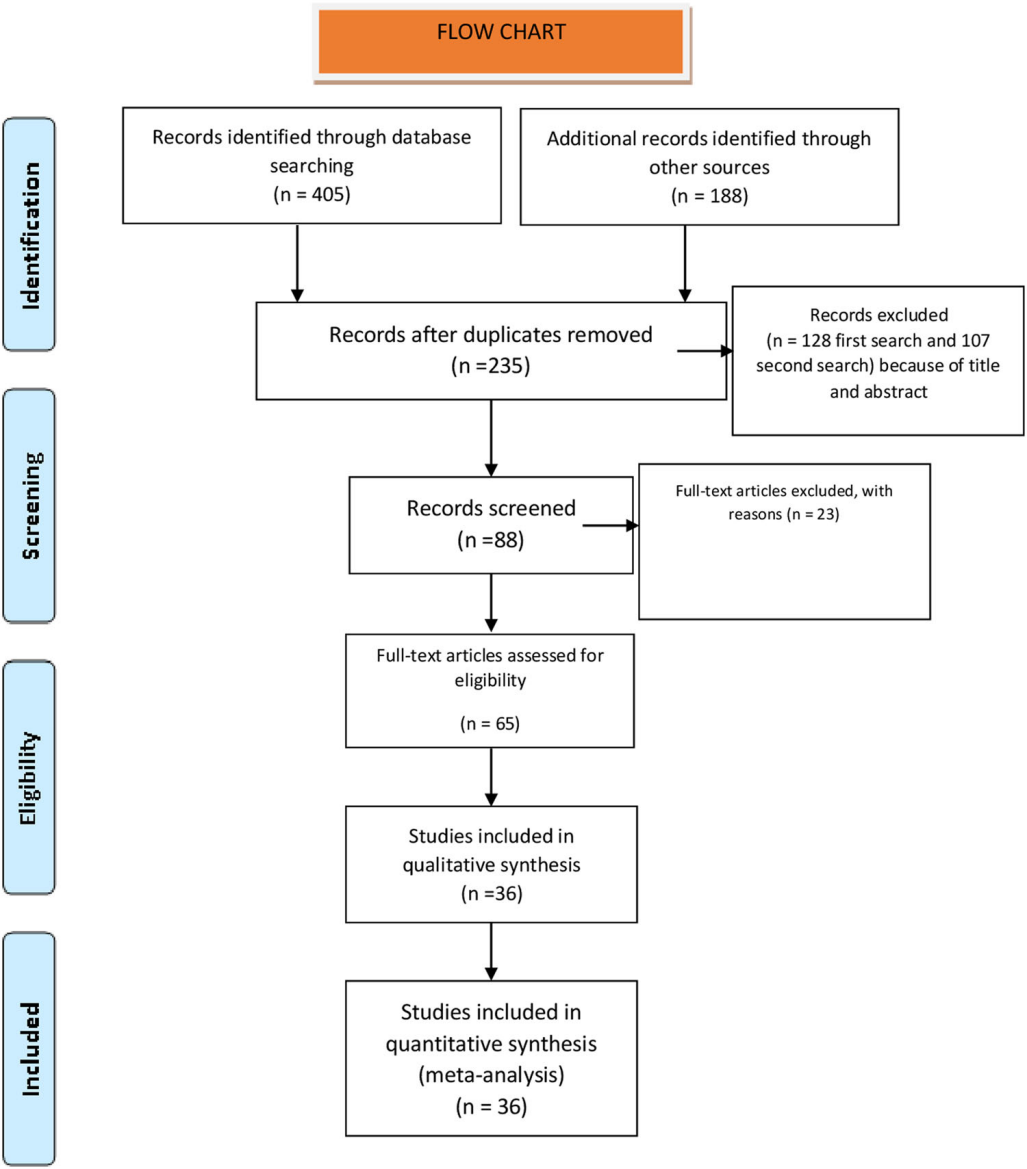
Flowchart

A systematic review was performed according to the preferred reporting items for systematic reviews and meta‐analyses statement. A search of the Web of Science, MEDLINE, and EMBASE databases was done with the search terms, “oral Wegener granulomatosis,” with studies after 1980 included where a consensus about the diagnosis of GPA was achieved and the c‐ANCA test was available. The MeSH terms were “oral granulomatosis with polyangiitis” AND “strawberry gingiva” AND “Wegener gingival hyperplasia.” The search was completed on January 10, 2022. The results were limited to human‐subject and English‐language articles. All abstracts were analyzed, and full‐text articles were obtained when inclusion criteria were fulfilled (Figure 1). Studies and publications with insufficient data or incomplete information were excluded. A manual search was also performed from the subsequent full‐text articles reviewed to identify additional relevant articles. Authors with manuscripts with relevant data and detailed information were contacted to obtain additional information. A protocol was enrolled and recorded with the International Prospective Register of Systematic Reviews (PROSPERO‐281771).

### 2.2 Selection criteria

Studies fulfilling inclusion criteria were observational studies designed (case series and case report). Studies with data about diagnosis, evolution, treatment, and follow‐up concerning WG or GPA were included. Exclusion criteria were non‐English language, animal, cadaveric, and radiologic studies, as well as letters to the editor. Nonobtainable full‐text studies, studies with insufficient data, review studies, and systematic reviews were also excluded. Studies with GPA located outside the mouth were also excluded. Two investigators (Alberto J. Peraza Labrador and Schillin W. Marchant) independently performed the search review and analyses. Any disagreements were resolved through discussion with all authors. The strength of evidence of the included articles was assessed with the Oxford Centre for Evidence‐Based Medicine classification system (Table 1). If data for any of the variables studied were missing, the Listwise deletion method was used. (When some cases had missing values of a particular variable; only cases with all or almost all variables in the analyses were used.) The quantitative analysis was performed by combining data in their original metric.

**Table 1:** Oxford centre for evidence‐based medicine classification system

Year	Author	Study Type	Cases (n)	Level of evidence
2000	Bergé S^20^	Case report	1	4
2009	Bhatt V^21^	Case report	1	4
1992	Eufinger H^22^	Case report	1	3b
2010	Barrett AW^23^	Case report	1	3a
2008	Hernandez G^24^	Case report	1	3b
1998	Lilly J^25^	Case report	1	4
2008	Carter LM^26^	Case report	1	3b
2009	Ruokonen H^27^	Case report	1	3b
1990	Cohen RE^28^	Case report	2	3b
2016	Hanisch M^29^	Case report	1	4
1993	Napier SS^30^	Case report	1	4
2017	Fonseca FP^31^	Case report	1	3b
2021	Kertesz T^32^	Case report	1	3a
2019	Patrick A^33^	Case report	1	4
2015	Sung IY^34^	Case report	1	4
2010	Reboll‐Ferrer RM^35^	Case report	1	4
2011	Siar CH^36^	Case report	1	3b
2018	Thompson G^37^	Case report	2	3b
2019	Msallem B^38^	Case report	1	3b
2020	Dhalkari CD^39^	Case report	1	4
2012	Heera R^40^	Case report	1	4
1994	Lustmann J^41^	Case report	1	4
1993	Vanhauwaert BG^13^	Case report	1	3b
1996	Ah‐See KW^42^	Case report	1	4
1981	Israelson H^43^	Case report	1	4
2014	Aravena V ^44^	Case report	1	4
2014	Genuis K^45^	Case report	1	3b
2011	Xing X^46^	Case report	1	3b
1985	Handlers JP^47^	Case report	1	3b
2012	Illes M^48^	Case report	1	4
2016	Brown P^49^	Case report	1	4
1993	Patten SF^1^	Case series	3	3b
1991	Allen CM^50^	Case series	3	3a
2020	Nico MMS^51^	Case series	4	3a
2007	Stewart C^5^	Case series	3	3a
2019	Szczeklik K^52^	Prospective	9	3a

## 3 DATA EXTRACTION

Variables included author, year of publication, study type, patient demographics, oral localization, salivary gland compromise, symptoms, diagnosis, treatment, and follow‐up. Data analyses were performed with Microsoft Excel 2018 (Microsoft Corp., Redmond, WA, USA).

## 4 DATA ANALYSIS

The analysis was performed using the Statistical Package for the Social Sciences (SPSS) software, version 20.0 Copyright IBM (SPSS Inc., Chicago, IL, USA).

## 5 RESULTS

A Medline, Web of Science, EMBASE, and ScienceDirect search was performed with 235 articles (Figure 1). A total of 31 case reports were included (Ah‐See et al., 1996; Allen et al., 1991; Aravena et al., 2014; Barrett et al., 2011; Bergé et al., 2000 Bhatt & Hall, 2009; Carter & Brizman, 2008; Cohen et al., 1990; Dhalkari et al., 2020; Eufinger et al., 1992; Fonseca et al., 2017; Genuis & Pewarchuk, 2014; Handlers et al., 1985; Hanisch et al., 2016; Heera et al., 2012; Hernández et al., 2008; Illes et al., 2012; Israelson et al., 1981; Kertesz et al., 2021; Lilly et al., 1998; Lustmann et al., 1994; Msallem et al., 2019; Napier et al., 1993; Patrick & Altman, 2019; Reboll‐Ferrer et al., 2010; Ruokonen et al., 2009; Siar et al., 2011; Sung et al., 2015; Thompson et al., 2018; Xing et al., 2011). Four case series (Allen et al., 1991; Nico et al., 2020; Patten and Tomecki, 1993; Stewart et al., 2007) and one prospective study (Szczeklik et al., 2019) were included in the analysis with an aggregate level of evidence 3b. The studies were from 16 different countries.

### 5.1 Demographics

*Sex*: Data were available for 52 patients, of whom 57.7% (*n* = 30) were females and 42.3% (*n* = 22) were males (Table 2).

**Table 2:** Demographic of GPA, race, type of lesion, oral lesions, salivary gland compromised, size, and evolution

	Female	Male	Total	
Total sample size	57.7% (*n* = 30)	42.3% (*n* = 22)	100% (*n* = 52)	CI
Age (years)	Mean ± SD (Min/Max)	Mean ± SD (Min/Max)	Mean ± SD (Min/Max)	[CI 95%]
	51.7 ± 14.12 (20/77)	46.7 ± 20.9 (6/87)	49.6 ± 17.3 (6/87)	[45–54.4]
*Race*	57.7% (*n* = 30)	42.3% (*n* = 22)	100% (*n* = 52)	[CI 95%]
White	63.3% (*n* = 19)	54.5% (*n* = 12)	59.6% (*n* = 31)	[46.3–72.9]
Brown	–	9.1% (*n* = 2)	3.8% (*n* = 2)	[−1.4 to 9]
Black	–	4.5% (*n* = 1)	1.9% (*n* = 1)	[−1.8 to 5.6]
No report	36.7% (*n* = 11)	31.8% (*n* = 7)	34.6% (*n* = 18)	NA
*Gingival/strawberry lesion*	57.7% (*n* = 30)	42.3% (*n* = 22)	100% (*n* = 52)	[CI 95%]
Yes	56.7% (*n* = 17)	68.2% (*n* = 15)	61.5% (*n* = 32)	[48.3–74.7]
No	30% (*n* = 9)	31.8% (*n* = 7)	30.8% (*n* = 16)	[18.3–43.3]
No report	13.3% (*n* = 4)	–	7.7% (*n* = 4)	NA
*Oral mucosa ulcer*				
Yes	23.3% (*n* = 7)	36.4% (*n* = 8)	28.8% (*n* = 15)	[16.5–41.1]
No	73.3% (*n* = 22)	59.1% (*n* = 13)	67.3% (*n* = 35)	[54.5–80.1]
No report	3.3% (*n* = 1)	4.5% (*n* = 1)	3.8% (*n* = 2)	NA
*Tooth mobility*				
Yes	16.7% (*n* = 5)	36.4% (*n* = 8)	25% (*n* = 13)	[13.2–36.8]
No	56.7% (*n* = 17)	36.4% (*n* = 8)	48.1% (*n* = 25)	[34.5–61.7]
No report	26.7% (*n* = 8)	27.3% (*n* = 6)	26.9% (*n* = 14)	NA
*Symptoms*				
Pain	56.7% (*n* = 17)	40.9% (*n* = 9)	50% (*n* = 26)	[36.4–63.6]
Painless lesion	16.7% (*n* = 5)	13.6% (*n* = 3)	15.4% (*n* = 8)	[5.6–25.2]
Bleeding	–	9.1% (*n* = 2)	3.8% (*n* = 2)	[−1.4 to 9]
No report	26.7% (*n* = 8)	36.4% (*n* = 8)	30.8% (*n* = 16)	NA
*Facial palsy*				
Yes	3.3% (*n* = 1)	–	1.9% (*n* = 1)	[−1.8 to 5.6]
No	80% (*n* = 24)	90.9% (*n* = 20)	84.6% (*n* = 44)	[74.8–94.4]
No report	16.7% ( *n*= 5)	9.1% (*n* = 2)	13.5% (*n* = 7)	NA
*Salivary gland compromise*				
No	76.7% (*n* = 23)	90.9% (*n* = 20)	82.7% (*n* = 43)	[72.4–93]
Yes	13.3% (*n* = 4)	9.1% (*n* = 2)	11.5% (*n* = 6)	[2.8–20.2]
No report	10% (*n* = 3)	–	5.8% (*n* = 3)	NA
	Mean ± SD (*n*)	Mean ± SD (*n*)	Mean ± SD (*n*)	[CI 95%]
Size of the lesion (cm)	1.6 ± 0.83 (*n* = 18)	1.7 ± 1.3 (*n* = 9)	1.7 ± 0.9 (*n* = 27)	[1.4–2]
Evolution time before diagnosis (months)	6.5 ± 12.2 (*n* = 21)	6.4 ± 10.5(*n* = 12)	6.4 ± 11.5 (*n* = 33)	[6.1–6.7]
Follow‐up (months)	27.5 ± 48.3 (*n* = 20)	16.5 ± 20.7 (*n* = 11)	23.6 ± 40.6 (*n* = 33)	[9.7–37.5]

Oral anatomic area	Female	Male	Total	CI
Upper gums	23.3% (*n* = 7)	36.5% (*n* = 8)	28.8% (*n* = 15)	[10.1–32.3]
Lower gums	13.3% (*n* = 4)	–	7.7% (*n* = 4)	[0.5–14.9]
Both at the same time	13.3% (*n* = 4)	27.3% (*n* = 6)	19.2% (*n* = 10)	[8.5–29.9]
Hard palate	20.7% (*n* = 6)	9.1% (*n* = 2)	15.3% (*n* = 8)	[1.6–17.6]
Oral mucosa	13.4% (*n* = 4)	27.4% (*n* = 6)	19.3% (*n* = 10)	[−3.6 to 22.2]
Tongue	9.4% (*n* = 3)	9.1% (*n* = 2)	9.7% (*n* = 5)	[1.6–17.6]
The lesion has not reported ubication	13.3% (*n* = 4)	4.3% (*n* = 1)	9.7% (*n* = 5)	[−1.8 to 10.6]
*Radiographic jaws image*				
Maxillary image	6.7% (*n* = 2)	–	3.8% (*n* = 2)	[−1.4 to 9]
Mandibular image	6.7% (*n* = 2)	9.1% (*n* = 2)	7.7% (*n* = 4)	[0.5–14.9]
Not lesion evident	20% (*n* = 6)	54.5% (*n* = 12)	34.6% (*n* = 18)	[21.7–47.5]
Both Jaws image	–	4.5% (*n* = 1)	1.9% (*n* = 1)	[−1.8 to 5.6]
Not reported	66.7% (*n* = 20)	31.8% (*n* = 7)	51.9% (*n* = 27)	NA
*Radiographic image outside jaws*				
Paranasal sinuses	20% (*n* = 6)	31.8% (*n* = 7)	25% (*n* = 13)	[13.2–36.8]
Lungs	13.3% (*n* = 4)	9.1% (*n* = 2)	11.5% (*n* = 6)	[2.8–20.2]
Lungs and paranasal sinuses	1.3% (*n* = 1)	–	1.9% (*n* = 1)	[−1.8 to 5.6]
Not reported	63.3% (*n* = 19)	59.1% (*n* = 13)	61.5% (*n* = 32)	NA
*GPA outside the oral cavity*				
Lungs	13.3% (*n* = 4)	13.5% (*n* = 3)	13.5% (*n* = 7)	[4.2–22.8]
Kidney	6.7% (*n* = 2)	–	3.8% (*n* = 2)	[−1.4 to 9]
Paranasal sinuses	10% (*n* = 3)	36.4% (*n* = 8)	21.2% (*n* = 11)	[10.1–32.3]
Lung and kidney	3.3% (*n* = 1)	13.6% (*n* = 3)	7.7% (*n* = 4)	[0.5–14.9]
Paranasal sinuses and lungs	13.3% (*n* = 4)	9.1% (*n* = 2)	11.5% (*n* = 6)	[2.8–20.2]
Sinus lungs and kidneys	6.7% (*n* = 2)	–	3.8% (*n* = 2)	[−1.4 to 9]
Skin	6.7% (*n* = 2)	4.5% (*n* = 1)	5.8% (*n* = 3)	[−0.6 to 12.2]
Not lesion evident	13.3% (*n*=4)	4.5% (*n* = 1)	9.6% (*n* = 5)	[1.6 to 17.6]
Not reported	26.7% (*n*=8)	18.2% (*n* = 4)	23.1% (*n* = 12)	NA
*Histology presentation*				
Inflammation, necrosis, and vasculitis	16.7% (*n* = 5)	36.4% (*n* = 8)	25% (*n* = 13)	[13.2–36.8]
Chronic inflammation, giant cells	50% (*n* = 15)	31.8% (*n* = 7)	42.3% (*n* = 22)	[28.9–55.7]
Acute inflammation	6.7% (*n* = 2)	4.5% (*n* = 1)	5.8% (*n* = 3)	[−0.6 to 12.2]
Granulomatous process	3.3% (*n* = 1)	4.5% (*n* = 1)	3.8% (*n* = 2)	[−1.4 to 9]
Histology not reported	23.3% (*n* = 7)	22.7% (*n*=5)	23.1% (*n* = 12)	NA
*c‐ANCA test*				
Not performed	10% (*n* = 3)	13.6% (*n* = 3)	11.5% (*n* = 6)	[2.8–20.2]
Performed	70% (*n* = 21)	72.7% (*n* = 16)	71.2% (*n* = 37)	[58.9–83.5]
Not reported	20% (*n* = 6)	13.6% (*n* = 3)	17.3% (*n* = 9)	NA
*Treatment*				
Prednisone + cyclophosphamide	50% (*n* = 15)	54.5% (*n* = 12)	51.9% (*n* = 27)	[38.3–65.5]
Prednisone + methotrexate	13.3% (*n* = 4)	22.7% (*n* = 5)	17.3% (*n* = 9)	[7–27.6]
Prednisone only	20% (*n* = 6)	18.4% (*n* = 4)	19.1% (*n* = 10)	[7–27.6]
Not specified therapy used	16.7% (*n* = 5)	4.5% (*n* = 1)	11.6% (*n* = 6)	[2.8–20.2]
*Patient's status*				
Dead	10% (*n* = 3)	–	5.8% (*n* = 3)	[−0.6 to 12.2]
Alive	86.7% (*n* = 26)	90.0% (*n* = 20)	88.5% (*n* = 46)	[79.8–97.2]
Not reported	3.3% (*n* = 1)	9.1% (*n* = 2)	5.8% (*n* = 3)	NA

*Note*: Characteristics of granulomatosis with polyangiitis, type of presentation and oral area, histological features, treatment, and patient's status.

Abbreviations: c‐ANCA, antineutrophil cytoplasmic autoantibody, cytoplasmic; CI, confidence interval; GPA, granulomatosis with polyangiitis; NA, not applicable.

*Age*: The average was 49.6 years, with a range from 6 to 87 years. The mean age for females was 51.7 years and for males 46.7 years (Table 2).

*Race*: Data were available for 34 patients, with white patients being the most common with 59.6% (*n* = 31), however 34.6% (*n* = 18) of the cases, did not report the race (Table 2).

*Strawberry gingivitis* (SG): Mentioned in 48 cases with 61.5% (*n* = 32) positive cases for this clinical presentation, SG was present in females 56.7% (*n* = 17), of the time, and in males 68.2% (*n* = 15). In 28.8% (*n* = 15) oral mucosal ulceration was present, and in 25% (*n* = 13) cases, tooth mobility was present (Table 2).

*Size*: Mentioned in 27 cases, with a mean of 1.7 cm; which was equal for both sexes.

*Symptomatology*: The information was found for 32 cases. Pain was the most common symptom with 50% (*n* = 26), for females 56.7% (*n* = 17) cases and for males 40.9% (*n* = 9), in 30.8% (*n* = 16), symptomatology was not reported (Table 2).

*Localization*: Mentioned in 43 cases; with the upper gums the most common site in 28.8% (*n* = 15), followed by both upper and lower gums at the same time with 19.2% (*n* = 10). Upper gingiva involvement in females was 23.3% (*n* = 7), and 36.5% (*n* = 8) for males, followed by maxillary and mandibular gingival lesions at the same time with 19.2% (*n* = 10). Interestingly, lesions localized to the mandibular gingiva were not found in males (Table 2).

*Salivary gland affected by GPA*: The information was available for 49 cases, where 11.5% (*n* = 6) of cases were positive for a lesion in the salivary gland. These lesions began after the oral mucosa was compromised. Facial palsy was found in one case 1.9% (*n* = 1) and was correlated with a salivary gland lesion (Table 2).

*Evolution time of the lesion before diagnosis*: The information was reported in 33 cases, with 6.4 months as the average evolution time; the average for females was 6.5 months and for males 6.4 months.

*Histopathology*: The information was found in 40 cases, where “chronic inflammation and giant cells” was the most common histologic presentation occurring in 42.3% (*n* = 22), followed by “inflammation, necrosis and vasculitis,” with 25% (*n* = 13). In 23.1% (*n* = 12) cases, histopathology was not reported (Table 2).

*c‐ANCA test*: The information was found for 43 cases, with a positivity rate of 71.2% (n = 37). For females, 70% (*n* = 21) had a positive c‐ANCA; in males, 72.7% (*n* = 16) were positive. 17.3% (*n* = 9) did not report results for this test (Table 2).

Compromised organ outside the oral cavity: The information was found for 40 cases, where 21.2% (*n* = 11) cases showed paranasal sinus involvement that started after the oral lesion. Lung involvement occurred in 13.5% (*n* = 7). There were 23.1% (n = 12) of cases that did not report this feature (Table 2).

*Concerning treatment*: The information was found for 41 cases. Prednisone + cyclophosphamide was the most commonly used therapy in 51.9% (*n* = 27), followed by prednisone only in 19.1% (*n* = 10) of cases (Table 2).

*Follow‐up*: The mean follow‐up for patients was found in 33 cases with a total of 23.6 months, for females at 27.5 months, and for males at 16.5 months (Table 2).

*Survival data*: Survival datawas found in 49 cases, with 88.5% (*n* = 46) alive at follow‐up, and 5.8% (*n* = 3) having died because of complications of the disease (Table 2).

## 6 DISCUSSION

GPA is an autoimmune disease of unknown etiology, the pathogenesis seems to involve hypersensitivity and an immune altered response mediated by humoral and cellular pathways (Ponniah et al., 2005). Given the rarity of this disease concerning oral lesions, the information in the literature has been limited to case reports, case series, and one prospective study. As a result, the epidemiology and oral molecular process of this disease remain undefined. In this systematic review, we aim to provide a more comprehensive characterization of oral GPA.

### 6.1 Clinical information

**Figure 2:**
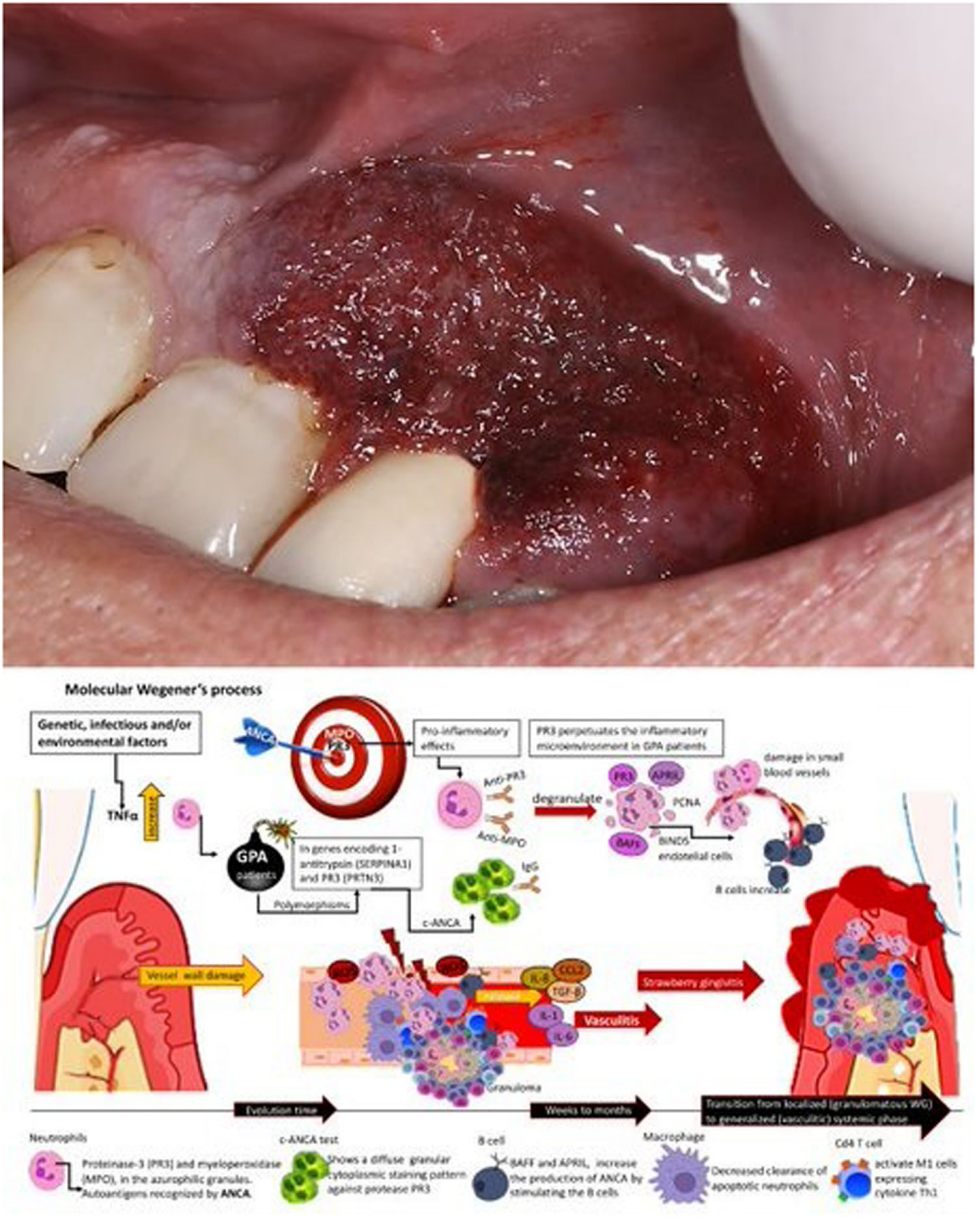
Legend clinical strawberry gingivitis and the molecular process. Genetic environmental factors and/or infectious (*Staphylococcus aureus*) increase the risk of granulomatosis with polyangiitis (GPA) to initiate or relapse. Overproduction of autoantibodies directed mainly against proteinase 3 (PR3‐ANCA). PR3 is expressed at the neutrophil surface, whereas myeloperoxidase is not. Increasing production of tumor necrosis factor (TNF‐α). Inducing expression of major histocompatibility complex, Class II, DP beta 1 (HLA‐DPB1) considerably more prevalent in patients with GPA. Also polymorphisms in the genes encoding 1‐antitrypsin (SERPINA1) and PR3 (PRTN3). This results in early neutrophil apoptosis which, combined with decreased clearance of apoptotic neutrophils by macrophages, induces the release of toxic mediators that convert the vessel inflammation to necrotizing vasculitis. Monocytes may also be activated by circulating ANCA, allowing granuloma formation. c‐ANCA, cytoplasmic antineutrophil cytoplasmic antibody; IgG, immunoglobulin G; IL‐1, interleukin‐1; PCNA, proliferating cell nuclear antigen.

The location and type of oral presentation vary in GPA patients; in our review, the 52 patients diagnosed with oral GPA were, on average 49.6 years with an age range of 6–87 years, relatively similar to Apoita‐Sanz et al. (2020) and Xing et al. (2011) with an average age of 47.4, in 20 cases, and different from Kertesz et al. (2021); which found an average of 44 years. Both of these studies assessed less than half the number of our cases. Regarding gender, a female predominance was found in 57.7% (*n* = 30), similar to previous studies (Ah‐See et al., 1996; Apoita‐Sanz et al., 2020; Aravena et al., 2014; Bhatt & Hall, 2009; Genuis & Pewarchuk, 2014; Handlers et al., 1985; Hernández et al., 2008; Raustia et al., 1985), but different from (Kertesz et al., 2021), who found a male predilection (male:female = 1.2:1). Concerning race, the most common was White patients 59.6% (*n* = 31), (Bergé et al., 2000; Bhatt & Hall, 2009; Genuis & Pewarchuk, 2014; Israelson et al., 1981; Kertesz et al., 2021; Msallem et al., 2019; Ruokonen et al., 2009; Stewart et al., 2007; Sung et al., 2015; Szczeklik et al., 2019; Thompson et al., 2018) The location of the lesion was more common for the upper gingiva for both sexes (Figure 2), but surprisingly lesions in the lower gingiva were not evident as a single lesion in male patients. The most common presentation of gingival lesions was strawberry‐like gingivitis with 61.5% (*n* = 32) cases.

According to Raustia et al. (1985), gingival GPA affects the interdental papillae most severely, a sign that was not very evident in our reviewed cases. In this regard, gingival GPA usually begins as localized involvement and may spread to the entire buccal and/or lingual gingival surfaces (Stewart et al., 2007). The second most common type of clinical lesion was mucosal ulceration 28.8% (*n* = 15), which was found more on the oral mucosa, tongue, and hard palate. As a consequence periodontal treatment does not seem to work for these patients (Fonseca et al., 2017). The mean tumor size found was 1.7 cm, with a range of 1.3–2 cm.

Concerning symptoms, the pain was the most common presentation in 50% (*n* = 26), followed by a painless lesion in 15.4% (*n* = 8). On the other hand, the radiographic features were nonspecific, although the bone loss was found in some cases that promoted tooth mobility, suggesting an advanced process (Bhatt & Hall, 2009; Cohen et al., 1990; Lilly et al., 1998; Msallem et al., 2019; Sivolella et al., 2012; Sung et al., 2015). Information on recurrence was not sufficiently reported, to allow conclusions to be reached in this review.

### 6.2 Evolution time before diagnosis

Our study found in 33 cases, an average of 6.4 months before oral GPA diagnosis, this was similar for both genders, 6.5 ± 12.2 months (*n* = 21) for females, and 6.4 ± 10.5 months (*n* = 12) for males. In a large study by Abdou et al. (2002), with 701 patients, only 22% of patients were diagnosed in the first month of illness, and 46% were diagnosed between 1 and 6 months after their initial symptoms. About 15% of patients recognized a delay of 6–12 months, and 18% a delay greater than 1 year, before definitive diagnosis. But this study was not only for oral GPA lesions, evidencing the necessity of a multidisciplinary group approach to establish a quick diagnosis.

### 6.3 ANCA test

This test became available in the mid‐1980s. Most patients with a c‐ANCA pattern have an ANCA directed against PR3, as determined by enzyme‐linked immunosorbent assay (Chen & Kallenberg, 2009). ANCA are autoantibodies directed against cytoplasmic constituents of neutrophils and monocytes. Most patients with active generalized GPA have c‐ANCA with PR3 specificity (Hagen et al., 1998). This PR‐3 is the antigen responsible for the immunostaining; nevertheless, the role in the pathogenesis of GPA is still unclear, an early c‐ANCA test may allow for earlier diagnosis, leading to more prompt therapy. However, the test sensitivity is known to be more accurate in the later stages of the disease. For systemic GPA, the sensitivity approaches 96%, while in localized forms, it is less than 67%. Still, our study found a positivity of 71.2% (*n* = 37), for oral lesions as a first clinical presentation, which is something to highlight in our analyses.

### 6.4 Histology

According to Devaney et al. (1998), GPA has three distinctive histopathological features: necrosis, granulomatous inflammation, and vasculitis, with multinucleated giant cells. However, when gingival biopsies are performed, the pathologist who does not find necrotizing granulomas and vasculitis or granulomatous vasculitis would find it nearly impossible to make a definitive diagnosis (Eufinger et al., 1992). The granulomatous lesions of GPA are composed of CD4+ T‐cells, CD8+ T‐cells, CD20+ B‐lymphocytes, neutrophil granulocytes, CD68+ macrophages, and CD68+ multinucleated giant cells (Figure 2). In our results in gingival biopsies, chronic inflammation and giant cells were the most common histologic presentation in 42.3% (*n* = 22), followed by inflammation, necrosis, and vasculitis, in 25% (*n* = 13). As an important feature, the delay in the definitive diagnosis was most common when chronic inflammation with giant cells was the histologic appearance on the initial biopsy. When the clinical presentation was oral ulcers, nonspecific inflammation was typically found. This finding is similar to. This can give a better understanding of how difficult it is to diagnose this disease with a histological study.

### 6.5 GPA outside the oral cavity

This study found 21.2% (*n* = 11) of cases, with lesions in the paranasal sinuses, compared with 15.3% (*n* = 8) presenting palatal lesions. However, there is a possibility in three palatal lesion cases that the disease started in the sinus and then reached the oral cavity, as a downward extension of GPA from the nose and nasal septum (Kasifoglu et al., 2008). On the other hand, eight cases could have started in the oral mucosa and subsequently spread to the sinus. None of the reported cases mentioned sinus symptoms, such as rhinorrhea, epiphora (Khan et al., 2006), purulent bloody nasal discharge, or chronic sinusitis (Apoita‐Sanz et al., 2020).

The second most commonly involved organ was the lung with 13.5% (*n* = 7) cases. However, there were cases of simultaneous sinus and lung involvement at 11.5% (*n* = 6), as well as lung and kidney at 7.7% (*n* = 4). The range of other organs/tissues involved in GPA is enormous and includes the musculoskeletal tissues, cutaneous tissues, neurological system, joint areas, and more (Apoita‐Sanz et al., 2020), which were not evident in the aforementioned cases.

Regarding radiographic evidence of involvement outside the oral cavity, in 20 cases, the GPA was evident in the paranasal sinus in 25% (*n* = 13), followed by the lungs in 11.5% (*n* = 6).

### 6.6 Treatment and patient outcome

The infrequency of Wegener's has hindered the formulation of a standardized treatment protocol. Most authors agree that corticoid therapy is the ideal treatment for this disease (Abdou et al., 2002), as most patients respond well to a high dose of steroids, cyclophosphamide, and/or azathioprine, with the possibility of long‐term remission (Tarabishy et al., 2010). In this study, the most common therapy used was prednisone with cyclophosphamide in 51.9% (*n* = 27). Prednisone alone was used in 19.1% (*n* = 10) cases, showing a fair response in follow‐up.

Immunosuppressive therapy has improved the 5‐year survival to 70%–80%; although, only 75% of patients achieve remission (Tarabishy et al., 2010). Relapses can appear in approximately 50% of patients and recurrence may be noted 18 months to 15 years following initial remission. Nevertheless, in our findings, the recurrence of oral lesions was less than 0.5%. In our analyzed studies the prognosis was excellent showing almost 94% survival, with only 5.8% cases (*n* = 3) dying from the disease. An important clinical finding was that these 3 patients each presented gingival strawberry lesions, tongue necrosis, and hard palate perforation.

### 6.7 Differential diagnosis

A principal differential diagnosis is a microscopic polyangiitis, defined as a systemic necrotizing vasculitis that clinically and histologically affects small‐sized vessels, without granulomata and that is associated with focal segmental necrotizing glomerulonephritis (Apoita‐Sanz et al., 2020). Other lesions in the differential diagnosis include drug‐induced gingival overgrowth, nonneoplastic proliferative lesions, Langerhan's cell histiocytosis, Kaposi's sarcoma, infections by *mycobacteria*, and metastatic disease, but these are considered less likely (Fonseca et al., 2017). Differentiation from ligneous gingivitis could also be necessary (Sivolella et al., 2012).

Our study had some limitations. First, the number of studies included was somewhat small. Second, a lack of complete data provided by each study limited the comprehensive studying of the demographics and time trends of the incidence of oral GPA. Third, available studies consisted only of published data. Unpublished data were not identified even though we tried to reach some of the authors. This suggests that publication bias cannot be absolutely excluded even though no significant publication bias was observed and analyzed with the Oxford codification system. It was impossible to completely exclude the influence of confounding factors inherent in these included studies. In this regard studies included in this systematic review were from a wide range of years, possibly making a point that treatment modalities have evolved over time. In addition, the cases were not homogenous. Despite these limitations, this study provides a comprehensive detailing of the literature, concerning this topic.

## 7 CONCLUSION

Oral GPA commonly presents with gingival strawberry‐like lesions, with pain, and affects white female patients most often. We found that the diagnosis was difficult to establish, a positive c‐ANCA test is critical, as is the histopathological study. If untreated the disease can be associated with substantial morbidity and mortality. Regarding the lack of GPA occurring as a single lesion in the lower gums in males, a more in‐depth analysis is necessary. For the oral clinician, this disease needs to be considered and addressed in the differential diagnosis of oral lesions. However, this study gives an update on the trends in demographics, treatment, and outcome for this uncommon disease.

## AUTHOR CONTRIBUTIONS

**Alberto J. Peraza Labrador**: Conceptualization; data analysis; and writing. **Luciano H. M. Valdez, Nestor R. Gonzalez Marin, Karem A. R. Ibazetta**: Methodology and writing – original draft. **Joan A. L. Chacón, Alberto J. V. Fernandez, and Katman B. T. Sanchez**: Validation; table and figure; and editing. **Cesar A. Villacrez and Schilin W. Marchant**: Software analyzes; Excel data; and references.

## CONFLICT OF INTEREST

The authors declare no conflict of interest.

## Supporting information

Supplementary information.

## Data Availability

No data were available.
