# Supplementary material for: Oral granulomatosis with polyangiitis a systematic review
Source: Clin Exp Dent Res. 2023 Jan 4;9(1):100–11. doi: 10.1002/cre2.706 (PMC9932239; doi:10.1002/cre2.706)
Supplement: Supplementary file 1 — Supplementary information. [file CRE2-9-100-s001.docx]

| **Year** | **Author** | **Study Type** | **Cases (n)** | **Level of evidence ^a^** |
| --- | --- | --- | --- | --- |
| 2000 | Bergé S ^20^ | Case report | 1 | 4 |
| 2009 | Bhatt V ^21^ | Case report | 1 | 4 |
| 1992 | Eufinger H ^22^ | Case report | 1 | 3b |
| 2010 | Barrett AW ^23^ | Case report | 1 | 3a |
| 2008 | Hernandez G ^24^ | Case report | 1 | 3b |
| 1998 | Lilly J ^25^ | Case report | 1 | 4 |
| 2008 | Carter LM ^26^ | Case report | 1 | 3b |
| 2009 | Ruokonen H ^27^ | Case report | 1 | 3b |
| 1990 | Cohen RE ^28^ | Case report | 2 | 3b |
| 2016 | Hanisch M ^29^ | Case report | 1 | 4 |
| 1993 | Napier SS ^30^ | Case report | 1 | 4 |
| 2017 | Fonseca FP ^31^ | Case report | 1 | 3b |
| 2021 | Kertesz T ^32^ | Case report | 1 | 3a |
| 2019 | Patrick A ^33^ | Case report | 1 | 4 |
| 2015 | Sung IY ^34^ | Case report | 1 | 4 |
| 2010 | Reboll-Ferrer RM ^35^ | Case report | 1 | 4 |
| 2011 | Siar CH ^36^ | Case report | 1 | 3b |
| 2018 | Thompson G ^37^ | Case report | 2 | 3b |
| 2019 | Msallem B ^38^ | Case report | 1 | 3b |
| 2020 | Dhalkari CD ^39^ | Case report | 1 | 4 |
| 2012 | Heera R ^40^ | Case report | 1 | 4 |
| 1994 | Lustmann J ^41^ | Case report | 1 | 4 |
| 1993 | Vanhauwaert BG ^13^ | Case report | 1 | 3b |
| 1996 | Ah-See KW ^42^ | Case report | 1 | 4 |
| 1981 | Israelson H ^43^ | Case report | 1 | 4 |
| 2014 | Aravena V ^44^ | Case report | 1 | 4 |
| 2014 | Genuis K ^45^ | Case report | 1 | 3b |
| 2011 | Xing X ^46^ | Case report | 1 | 3b |
| 1985 | Handlers JP ^47^ | Case report | 1 | 3b |
| 2012 | Illes M ^48^ | Case report | 1 | 4 |
| 2016 | Brown P ^49^ | Case report | 1 | 4 |
| 1993 | Patten SF ^1^ | Case series | 3 | 3b |
| 1991 | Allen CM ^50^ | Case series | 3 | 3a |
| 2020 | Nico MMS ^51^ | Case series | 4 | 3a |
| 2007 | Stewart C ^5^ | Case series | 3 | 3a |
| 2019 | Szczeklik K ^52^ | Prospective | 9 | 3a |
